# White Matter Abnormalities in Major Depression: A Tract-Based Spatial Statistics and Rumination Study

**DOI:** 10.1371/journal.pone.0037561

**Published:** 2012-05-30

**Authors:** Nianming Zuo, Jiliang Fang, Xueyu Lv, Yuan Zhou, Yang Hong, Tao Li, Haibing Tong, Xiaoling Wang, Weidong Wang, Tianzi Jiang

**Affiliations:** 1 LIAMA Center for Computational Medicine, National Laboratory of Pattern Recognition, Institute of Automation, Chinese Academy of Sciences, Beijing, China; 2 Laboratory for Functional Brain Imaging, Department of Radiology, Guang-an-men Hospital, China Academy of Chinese Medical Sciences, Beijing, China; 3 Department of Psychology, Guang-an-men Hospital, China Academy of Chinese Medical Sciences, Beijing, China; 4 Key Laboratory of Behavioral Science, Institute of Psychology, Chinese Academy of Sciences, Beijing, China; 5 Key Laboratory for NeuroInformation of Ministry of Education, School of Life Science and Technology, University of Electronic Science and Technology of China, Chengdu, China; 6 The Queensland Brain Institute, The University of Queensland, Brisbane, Queensland, Australia; Bellvitge Biomedical Research Institute-IDIBELL, Spain

## Abstract

Increasing evidence indicates that major depressive disorder (MDD) is usually accompanied by altered white matter in the prefrontal cortex, the parietal lobe and the limbic system. As a behavioral abnormity of MDD, rumination has been believed to be a substantial indicator of the mental state of the depressive state. So far, however, no report that we are aware of has evaluated the relationship between white matter alterations and the ruminative state. In this study, we first explored the altered white matter using a tract-based spatial statistics (TBSS) method based on diffusion tensor imaging of 19 healthy and 16 depressive subjects. We then investigated correlations between the altered white matter microstructure in the identified altered regions and the severity of ruminations measured by the ruminative response scale. Our results demonstrated altered white matter microstructure in circuits connecting the prefrontal lobe, the parietal lobe and the limbic system (p<0.005, uncorrected), findings which support previous research. More importantly, the result also indicated that a greater alteration in the white matter is associated with a more ruminative state (p<0.05, Bonferroni corrected). The detected abnormalities in the white matter should be interpreted cautiously because of the small sample size in this study. This finding supports the psychometric significance of white matter deficits in MDD.

## Introduction

Major depressive disorder (MDD) is usually characterized by a low mood, a feeling of sadness and helplessness, and a loss of interest in pleasurable and formerly enjoyed activities [1]. Psychologically, various symptoms characterize depressive syndromes, including negative thoughts, pessimistic mood, self-injury intention etc. Measures of rumination are primarily utilized to assess the behavior of negative thoughts and to measure the intensity of memory rehearsal [2], [3], [4], [5]. The three dimensions of the measure (depressive symptoms, brooding and reflection) characterize different reactions of the depressive state to a negative cognitive bias [2], [6]. A number of research studies have investigated manifestations of rumination, such as behavioral performance [7], [8] and the role of the default-mode network [9], [10], in the evolution of MDD [11].

In recent years, investigations of anatomical and functional regions of the brain have built up a picture of the structural changes in the cerebral cortex and white matter fibers associated with MDD [12], [13], including changes in areas such as the prefrontal cortex [2], [14], [15], [16], the temporal cortex and limbic system [16], [17], [18], [19], the internal capsule [14], the uncinate fasciculus [20], [21] and the superior longitudinal fasciculus [1], [21], [22]. By combining the locations of the above neurodegenerated regions with information from relevant animal experiments [23], a frontal-limbic circuit abnormality has been hypothesized as the locus of the dysfunction underlying mood-regulation in MDD [23], [24]. The frontal-limbic system is the circuit critical to executive function, cognitive control and emotion regulation; and its dysregulation is usually related to psychiatric disorders [25], including major depression [12], [18], bipolar depression [26], [27] and schizophrenia [28]. In order to more precisely elucidate the interactions between the static microstructural lesions and the dynamic dysfunctions manifested as behavioral performance in MDD [1], several research studies have exploited various behavioral measurements to investigate the relationship between white matter abnormalities and the mental state, including measurements based on the Bech–Rafaelsen Melancholia Scale [29] and the Hamilton Depression Rating Scale (HAM-D) [30]. However, the relationship between the depressive syndrome, as characterized by rumination measures, and the altered brain circuit in MDD remains unclear.

Since its emergence two decades ago, diffusion tensor MRI (DTI) has become a promising technique for characterizing alterations of white matter fibers *in vivo* [31], [32]. To visualize a neural path, DTI uses a mathematical model (such as a Gaussian model), by which several attributes are extracted from the tensor to characterize the diffusion properties, and hence the structure, of white matter. The most commonly used measure is fractional anisotropy (FA), which is used to elucidate the structural organization of the neural fibers [33], [34]. Based on the FA map extracted through DTI, Smith and colleagues [35] proposed a tract-based spatial statistics (TBSS) approach that conducts statistical analysis along individual white matter skeletons. The construction of an individual skeleton will mitigate most misalignments introduced by co-registration between the subjects and will improve the ability to localize abnormalities [34], [35]. DTI-based studies of MDD have reported altered white matter microstructure in the frontal-limbic circuit, in compliment to other findings previously described [12], [13], [34].

In the present study, we acquired DTI image data from a group of young depressed and healthy adults and explored the abnormalities along the white matter skeletons using TBSS. We further investigated the relationships between the white matter lesions we identified by this method and clinical rumination measurements. We hypothesized that the severity of the ruminative state would correlate with the extent of the alterations of the mood-regulating circuits.

## Methods

### Participants

In this study, we recruited 16 depressed patients (age  = 37±9.4 years) from the Guang-an-men Hospital in Beijing. All the candidates were interviewed using the Structured Clinical Interview for DSM-IV. We also recruited 19 matched controls (age = 36.6±7.7 years) from the community (Table 1).

**Table 1:** Characteristics of the participants.

		Control (n = 19)	Depression (n = 16)	p-value
Gender^a^		7(M), 12(F)	3(M), 13(F)	0.24
Age (years)^b^		36.6±7.7	37±9.4	0.9
HAM-D^b^		3.0±2.0	30.3±6.2	1.0e-18
Rumination^b^		29.3±6.4	45.9±11.2	4.2e-6
	Depressive Symptoms^b^	14.9±2.6	24.4±6	6.6e-7
	Reflection^b^	7.2±2.1	9.9±3.7	0.01
	Brooding^b^	7.2±2.1	11.6±2.7	4.1e-6

a Chi-square was used for gender comparisons.

b Two sample two-tailed *t*-test was used for age and neuropsychological tests comparisons between the MDD and NC groups.

The last three rows are separately the three dimensions of rumination.

All the candidates were screened for craniocerebral trauma, hyperintension and other types of neurologic syndrome. Each subject completed the rumination questionnaire test with 21 items (RRS-21, Rumination Responses Scale, removed item 14 for Chinese candidates) [4], [6]. This experiment was approved by the Institutional Review Board of the Guang-an-men Hospital, and all participants provided a written consent form.

The recruited MDD patients fulfilled the following inclusion criteria: (1) aged in 20–50, in line with the ICD-10 diagnostic standard, (2) 24-item HAM-D measurement, for depressed >20 and for control <10, (3) no less than two weeks clearance for any medicine related to MDD or other psychiatric disorder, (4) education level greater than 12 years, (5) no less than two weeks since the onset of the current episode, (6) right-handed, (7) eligible for MRI scanning.

Candidates could not have (1) schizophrenia, medicine/alcohol addiction, (2) craniocerebral trauma, (3) gestation or lactation, (4) mania or related symptoms, (5) comorbidity of other mental disorders since the onset of depression, (6) completed electroconvulsive therapy.

### DTI Data Collection

DTI data was acquired in the Guang-an-men Hospital using a 1.5T GE SIGNA EXCITE scanner and a standard brain coil. The scanned FOV  = 26 cm×26 cm, and the exported image matrix was 256×256×35 and TR/TE  = 10000/96.2 ms, with a voxel size  = 1 mm×1 mm×4 mm, where the additional gap between sections was 0.5 mm. We collected data in 25 non-collinear directions with a diffusion intensity b = 900 s/mm^2^. We repeated the collection twice, namely NEX  = 2, averaging the data to reduce noise. The T2-weighted echo-planar images (EPI) were b_0_ = 0 s/mm^2^.

### Data Preprocessing

We used the FSL package (www.fmrib.ox.ac.uk/fsl) [36], [37] to preprocess the diffusion MRI data. First, we used the FSL/eddy_correct tool, which registers all the diffused images to the b_0_ image space, to correct for eddy currents and FSL/bet2 to skull-strip the brain using a threshold of 0.15. Finally, we used the FSL/dtifit tool to fit the diffusion tensors and to obtain the FA image map, which were traditionally defined as in [31], for each subject.
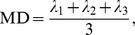


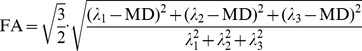



### Tract-based Spatial Statistics (TBSS)

We used the TBSS method [35] to explore group differences between the white matter skeletons derived from the FA images. First, the individual FA images from both the healthy and depressed subjects were each nonlinearly aligned to the pre-defined FSL FMRIB58 FA map using a resolution of 1 mm in the standard MNI152 space. Then, a mean FA image was created and thinned to create a mean FA skeleton which represented the centers of all tracts common to the group (http://fsl.fmrib.ox.ac.uk/fsl/tbss). Each subject’s aligned FA image was projected onto the above skeleton. Finally, voxel-wise statistical analyses were performed across the subjects for each point on the common skeleton. We performed a permutation test on the final skeleton to detect white matter differences between the depressed and the control group using the FSL/RANDOMISE tool (5000 permutations). Subsequently, we used the FSL/TBSS package to position the ROIs that elucidate the FA differences between the depressed and the comparison subjects on the white matter skeletons, using a significance level p<0.005 and a cluster size>60 voxels. We chose these parameters because using a lower level threshold yielded low confidence ROIs that were scattered throughout the entire brain rather than the few high confidence, localized ROIs that we identified using the higher threshold. However, if we had used too high a threshold, the small ROIs that would have been identified could have been susceptible to contamination by noise.

Finally, we investigated the relationship between the average FA value inside the ROIs and clinical mental measurements, including HAM-D and rumination scores. The average FA value and the clinical measurements were analyzed using an unpaired two-sample two-tailed *t*-test, with p<0.05 as the confidence threshold. Meanwhile, in order to remove age and gender as factors in the correlations, we repeated the experiments with and without regressing age and gender effects. Additionally, based on the above correlation results, we further conducted correlation analyses between the HAM-D scale and rumination and its subscales, to confirm whether the rumination effects could actually be dissociated from more general effects.

## Results

In this section, all results are presented in MNI-1 mm space, based on the standard FA template image in the FSL package.

**Figure 1:**
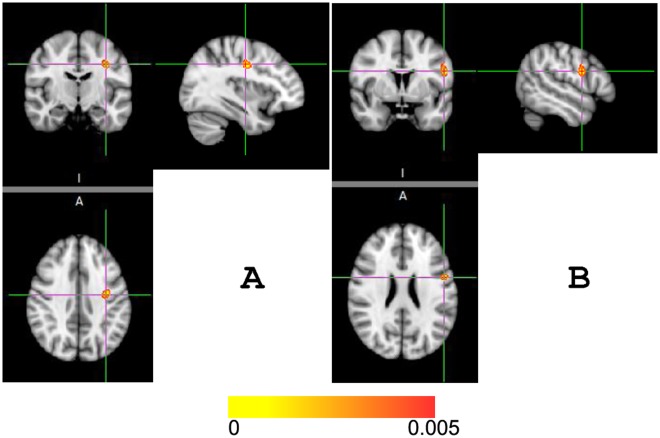
The anatomical locations of the ROIs derived by the TBSS method. They are separately located at the left central portion of the superior longitudinal fasciculus (SLF) (A) and the left inferior portion of the frontal lobe (IFL) (B).

We found two areas of significant alterations on the FA skeleton (p<0.005, uncorrected) (Fig. 1), in the left center portion of the superior longitudinal fasciculus (SLF) (65 voxels centering at [−37, −7, 33] in MNI coordinates) and in the premotor area (BA 6) (66 voxels centering at [−53, 3, 18] in MNI coordinates). In these areas the FA values in the healthy were greater than in the depressed. Table 2 lists comparisons of the average FA values between the healthy and depressed subjects for both of these ROIs and shows that the average FAs in the ROIs of the depressed were significantly altered compared with those in the healthy controls.

**Table 2:** Comparisons of the average FA values in the two ROIs between the healthy and depressed subjects.

ROI	Mean FA (±SD) Control	Mean FA (±SD) Depression	p-value(Control – Depression)
ROI in SLF	0.3656±0.0291	0.3277±0.0251	2.6813e-4
ROI in premotor area	0.2692±0.0348	0.2206±0.0322	1.6649e-4

**Figure 2:**
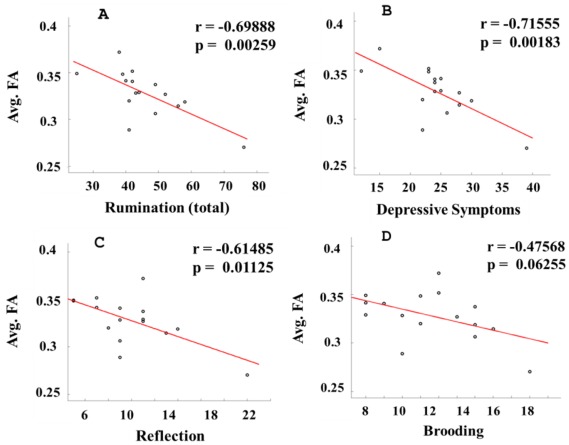
For the ROI in the left central portion of the superior longitudinal fasciculus (See Fig. 1A) in the depressive group, the average FA values are correlated with the rumination measurement and its sub-dimensions. (A) rumination (total) vs. average FA; (B) depressive symptoms vs. average FA; (C) reflection vs. average FA; (D) brooding vs. average FA. Avg: Average.

The average FA value for the ROI in the left center portion of the SLF in the frontal lobe (Fig. 1A) showed a strong negative correlation with the rumination score and its two sub-dimensions, depressive symptoms and reflection (p<0.05) (Fig. 2). All correlations, except for those for the brooding dimension, survived the Bonferroni correction (p = 0.05/4 = 0.0125) for multiple comparisons. We did not find significant correlations between the HAM-D and the rumination/subscales (*p*>0.16), and when we regressed out the HAM-D factors during the correlation analysis between rumination and its subscales and the mean FA, the results were extremely similar to those if we left the HAM-D factors in. In addition, when we studied the correlations under both conditions with and without regressing out age and gender as covariates, we consistently generated similar patterns of results (Table 3). The average FA for this ROI did not have a strong correlation with the rumination scales of the healthy group. Also, we did not detect any correlation between the HAM-D and rumination and its subscales (Table 4).

**Table 3:** Correlations between the average FA value in the ROI located in the center of the SLF and the associated rumination scales.

	Rumination		Depressive Symptoms		Reflection		Brooding	
	R	p	r	p	r	p	r	p
No Control	−0.69885	0.00259	−0.71545	0.00183	−0.61473	0.01128	−0.47553	0.06265
Control	−0.69888	0.00259	−0.71555	0.00183	−0.61485	0.01125	−0.47568	0.06255

“Control” and “No Control” refer to correlations with and without correction for gender and age factors.

**Table 4:** Correlation analysis between the HAM-D scales and rumination and its subscales in the depressed group.

HAM-D	Ruminationp/R	Depressivesymptomsp/R	Broodingp/R	Reflectionp/R
	0.7528/−0.0855	0.9658/0.0117	0.6705/0.1154	0.1668/−0.3632

We did not find significant correlations between the average FA and the associated rumination scores for the ROI in the left premotor area (Fig. 1B) in our MDD group. Additionally, for both ROIs, we found no significant correlation between the HAM-D scale and the average FA value in the MDD patients.

## Discussion

Based on DTI images for a group of young adults with MDD and a group of normal controls, we found decreased FA values in the center of the left superior longitudinal fasciculus (SLF), and in the premotor area (BA 6) (p<0.005, uncorrected) in the MDD group compared to the controls. Additionally, we found the scores for the ruminative state and its sub-scales were significantly negatively correlated to the FA values in the left SLF in the MDD patients. To our knowledge, this is the first study to examine the relationship between white matter alterations and the ruminative state that is believed to be relevant feature of MDD in young adults.

The FA index extracted from diffusion tensor imaging delineates the directional alignment of neural fibers. When neural fibers are longitudinally aligned or intensively myelinated, the FA is much stronger [26]. Based on this, we were able to derive from the current study that the depressive subjects have altered white matter microstructure in the center of the left SLF and in the left premotor area. Additionally, we also found that, in the depressive group, the greater number of altered fibers in the left SLF were associated with much higher levels of both rumination and its three sub-scales, namely depressive symptoms, reflection and brooding. In recent years, although numerous studies have reported altered white matter in geriatric MDD [2], [14], [19], [38], similar research in young adults and adolescence has received less attention [1]. Only a few DTI studies have reported preliminary evidence of alterations of the white matter in younger, depressive patients [1], [16], [21], [25], [39], [40], [41].

A variety of studies have reported altered white matter microstructure in the SLF of MDD patients [1], [18], [22], [29], and our results provided further evidence for this prevailing finding. The SLF is composed of several subtypes, including SLF I/II/III and the arcuate fascicle, and each region is engaged in distinct behavioral and cognitive functions [42], [43]. The SLF is a pivotal connection in the frontal, parietal, temporal and limbic circuits, which play a critical role in executive control, cognitive process and emotional modulation [12], [24], [44]. Specifically, SLF II is the major component of the SLF and originates in the caudal-inferior parietal cortex and terminates in the dorsal lateral prefrontal cortex (Brodmann 6, 8 and 46). The dorsal lateral prefrontal cortex is regarded as a core node in executive functioning [14], [45], and a number of functional imaging studies have reported its interactions with the limbic system [3], [46]. Therefore, as the major path connecting the frontal and parietal circuits and the limbic system, an impaired SLF would inevitably degrade its mediating role in mood regulation.

The specified ROI is located in a superficial area in the central part of the left SLF. It also contains fibers connecting the dorsal/ventral prefrontal cortex. In combination with the second ROI located in the premotor cortex, it composes a motor/sensory network that plays an important role in regulating motor, cognitive, and motivational processes [12], [45], [47]. Reports of altered premotor areas exist in MDD [48], [49], [50]. Furthermore, the use of several intervention methods, including rTMS (repetitive transcranial magnetic stimulation) [51], [52] and adjunctive use of anti-depressant medicine [53], on depressed patients also obviously enhanced the motor/sensory network at the same time that the depressive symptoms decreased. In a comparison with fluoxetine (an anti-depressant medicine), even the placebo effect enhanced the described motor/sensory network [54]. This partially shows a very strong interrelationship between reward/emotion regions and motor regions in modulating mental state [55], [56], [57], [58]. The premotor area acts in conceiving and integrating movement, functions which are believed to help regulate posture by dictating an optimal position for any given movement to the motor cortex [59]. Thus, the altered FA in the depressed patient may possibly address why the depressed are usually troubled by psychomotor retardation and executive function impairments [24], [60], [61], [62].

A further analysis of the correlation between the integrity of the SLF and the ruminative state also elucidates the story behind MDD. In the MDD group, the average FA values in the center of the SLF exhibited a strong negative correlation with the severity of the ruminative state (p<0.05, Bonferroni corrected). The correlation between the brooding component and FA also has an obvious negative trend (r = −0.47568, p = 0.06255). Gender and age factors did not have a significant influence on the correlation results. No correlation was found between the HAM-D scale and rumination or its subscales. This implies that more seriously impaired mood regulating circuits may be associated with deeper rumination in MDD. Depressive symptoms, reflection and brooding, are regarded as three relatively separate aspects describing the state of rumination and are believed to be general symptoms of mood disorders. They have been found to partially or fully mediate the relationship between depression and negative inferential styles and self-referential processing [2], [6], [63]. Reflective pondering is thought to deteriorate concurrently with the depressed state, but separately from depressive symptoms, by intensively reflecting on the cause of the low mood [6], [64]. Compulsive brooding is characterized as insistent self-criticizing in a negative environment and has been related to a more severe depressive state in both concurrent and longitudinal analyses [6], [64]. The strong negative correlations between the integrity of white matter fibers in the mood regulating path and the severity of the ruminative state also supports the hypothesis that MDD deteriorates the mood-regulated circuits [12], [13], [24], [63]. Several other studies based on different performance scales have also obtained similar correlations [19], [29], and our study further strengthens this position. Additionally, we could not determine the presence of a causal relationship between the depressed state and alterations of the white matter microstructure.

Unlike rumination and its subscales, we did not find any correlation between the FA values and the HAM-D score at either the SLF or the premotor areas. This can be addressed by considering the difference in the role of the HAM-D scale and that of the rumination scale. Although the rumination scale and the HAM-D scale both delineate depressive symptoms, they actually describe two different aspects of depression. To some extent, HAM-D can be regarded as measuring the severity of the depressive state, whereas rumination scales (using the ruminative response style (RRS) in this study) depict ruminative states that have the potential to intensify and possibly prolong a current depressive episode or even induce a new depressive episode [65], [66]. Therefore, occasionally a rumination scale and the HAM-D scale show similar trends in the same test situation, but this fact does not signify that they always have a positive correlation with each other. Some existing studies report possible negative correlations between FA values and the severity of symptoms, as characterized separately by the HAM-D, the BDI (Beck Depression Inventory [67]) and the MES (Bech–Rafaelsen Melancholia Scale [68]) [69], [70]. In addition, Nobuhara *et al.* [19] emphasized that, in most of the regions they detected, the FA lacked significant correlations with the severity of depressive symptoms as specified by the HAM-D. Even their remaining correlation was not strongly significant (*r* = −0.58, *p* = 0.04), but they did not correct the result for multiple tests. Similarly, we also did not detect significant correlations of the mean FA with the HAM-D scale for either of the ROIs in the depressed group. Another possible explanation for our failure to find a correlation is that the HAM-D scores in our depressed sample were concentrated around 30.3 (see Table 1 in the manuscript). When scores are tightly clustered in this way, they cannot contribute much to the linear regression. We thus were unable to reach any conclusion about whether such a correlation actually exists.

Although we found altered FA in the premotor area, we did not find a clear correlation between the altered FA and rumination and its subscales. We do not have a clear explanation for this, but it may have been due to drawbacks in our current sample, including the small sample size, different durations of depression, unbalanced gender ratios between the controls and the patients, etc. These potentially complicating factors should be further investigated in future studies.

The findings presented here should be interpreted cautiously, although they are in line with prevailing findings and provide alternative support to the dysregulating theory of the frontal-limbic circuit in MDD. That the detected ROIs could not survive the FDR correction may be ascribed to the small sample size, but the findings substantially exhibit obvious trends connected with white matter lesions in an MDD population. Furthermore, the patients recruited for this study were not strictly limited to those who were in a first episode of MDD, and some depressed candidates had endured a variety of anti-depression treatments. Although we required a two-week clearance of affective-related medications, we cannot exclude the possibility of a medication interaction with our results [30], [71].

In summary, our exploratory investigations demonstrate alteration of the white matter located in the neural circuit of the frontal-limbic system in MDD. Notably, the severity of the deterioration was significantly correlated with the presence of ruminative thought. Recognizing this relationship may help to pave the way for precise biomarkers of the evolution of MDD [72], [73] as well as providing a better assessment measure for clinical diagnosis and therapy.

## References

[1] Korgaonkar MS, Grieve SM, Koslow SH, Gabrieli JD, Gordon E (2010). Loss of white matter integrity in major depressive disorder: Evidence using tract-based spatial statistical analysis of diffusion tensor imaging.. Hum Brain Mapp.

[2] Taylor WD, MacFall JR, Payne ME, McQuoid DR, Provenzale JM (2004). Late-life depression and microstructural abnormalities in dorsolateral prefrontal cortex white matter.. Am J Psychiatry.

[3] Hooley JM, Gruber SA, Parker HA, Guillaumot J, Rogowska J (2009). Cortico-limbic response to personally challenging emotional stimuli after complete recovery from depression.. Psychiatry Res.

[4] Yang J, Ling Y, Xiao J, Yao S-q (2009). The Chinese Version of Ruminative Responses Scale in High School Students:Its Reliability and Validity (In Chinese).. Chinese Journal of Clinical Psychology.

[5] McLaughlin KA, Nolen-Hoeksema S (2011). Rumination as a transdiagnostic factor in depression and anxiety.. Behav Res Ther.

[6] Treynor W, Gonzalez R, Nolen-Hoeksema S (2003). Rumination Reconsidered: A Psychometric Analysis.. Cognit Ther Res.

[7] Thompson RJ, Mata J, Jaeggi SM, Buschkuehl M, Jonides J (2010). Maladaptive coping, adaptive coping, and depressive symptoms: variations across age and depressive state.. Behav Res Ther.

[8] Chan S, Miranda R, Surrence K (2009). Subtypes of rumination in the relationship between negative life events and suicidal ideation.. Arch Suicide Res.

[9] Berman MG, Peltier S, Nee DE, Kross E, Deldin PJ (2011). Depression, rumination and the default network.. Soc Cogn Affect Neurosci.

[10] Hamilton JP, Furman DJ, Chang C, Thomason ME, Dennis E (2011). Default-mode and task-positive network activity in major depressive disorder: implications for adaptive and maladaptive rumination.. Biol Psychiatry.

[11] Joormann J, Engle RW, Sedek G, von Hecker U, McIntosh DN (2005). Inhibition, rumination, and mood regulation in depression.. Cognitive Limitations in Aging and Psychopathology: Attention, Working Memory, and Executive Functions.

[12] Sexton CE, Mackay CE, Ebmeier KP (2009). A systematic review of diffusion tensor imaging studies in affective disorders.. Biol Psychiatry.

[13] Drevets WC, Price JL, Furey ML (2008). Brain structural and functional abnormalities in mood disorders: implications for neurocircuitry models of depression.. Brain Struct Funct.

[14] Bae JN, MacFall JR, Krishnan KR, Payne ME, Steffens DC (2006). Dorsolateral prefrontal cortex and anterior cingulate cortex white matter alterations in late-life depression.. Biol Psychiatry.

[15] Alexopoulos GS, Kiosses DN, Choi SJ, Murphy CF, Lim KO (2002). Frontal white matter microstructure and treatment response of late-life depression: a preliminary study.. Am J Psychiatry.

[16] Ma N, Li L, Shu N, Liu J, Gong G (2007). White matter abnormalities in first-episode, treatment-naive young adults with major depressive disorder.. Am J Psychiatry.

[17] Kieseppa T, Eerola M, Mantyla R, Neuvonen T, Poutanen VP (2010). Major depressive disorder and white matter abnormalities: a diffusion tensor imaging study with tract-based spatial statistics.. J Affect Disord.

[18] Cullen KR, Klimes-Dougan B, Muetzel R, Mueller BA, Camchong J (2010). Altered white matter microstructure in adolescents with major depression: a preliminary study.. J Am Acad Child Adolesc Psychiatry 49: 173–183.

[19] Nobuhara K, Okugawa G, Sugimoto T, Minami T, Tamagaki C (2006). Frontal white matter anisotropy and symptom severity of late-life depression: a magnetic resonance diffusion tensor imaging study.. J Neurol Neurosurg Psychiatry.

[20] Taylor WD, MacFall JR, Gerig G, Krishnan RR (2007). Structural integrity of the uncinate fasciculus in geriatric depression: Relationship with age of onset.. Neuropsychiatr Dis Treat.

[21] Huang H, Fan X, Williamson DE, Rao U (2011). White matter changes in healthy adolescents at familial risk for unipolar depression: a diffusion tensor imaging study.. Neuropsychopharmacology.

[22] Zou K, Huang X, Li T, Gong Q, Li Z (2008). Alterations of white matter integrity in adults with major depressive disorder: a magnetic resonance imaging study.. J Psychiatry Neurosci.

[23] Welch JM, Lu J, Rodriguiz RM, Trotta NC, Peca J (2007). Cortico-striatal synaptic defects and OCD-like behaviours in Sapap3-mutant mice.. Nature.

[24] Clark L, Chamberlain SR, Sahakian BJ (2009). Neurocognitive mechanisms in depression: implications for treatment.. Annu Rev Neurosci.

[25] Zhu X, Wang X, Xiao J, Zhong M, Liao J (2011). Altered white matter integrity in first-episode, treatment-naive young adults with major depressive disorder: a tract-based spatial statistics study.. Brain Res.

[26] Versace A, Almeida JR, Hassel S, Walsh ND, Novelli M (2008). Elevated left and reduced right orbitomedial prefrontal fractional anisotropy in adults with bipolar disorder revealed by tract-based spatial statistics.. Arch Gen Psychiatry.

[27] Blumberg HP, Krystal JH, Bansal R, Martin A, Dziura J (2006). Age, rapid-cycling, and pharmacotherapy effects on ventral prefrontal cortex in bipolar disorder: a cross-sectional study.. Biol Psychiatry.

[28] Farrow TF, Whitford TJ, Williams LM, Gomes L, Harris AW (2005). Diagnosis-related regional gray matter loss over two years in first episode schizophrenia and bipolar disorder.. Biol Psychiatry.

[29] Dalby RB, Frandsen J, Chakravarty MM, Ahdidan J, Sorensen L (2010). Depression severity is correlated to the integrity of white matter fiber tracts in late-onset major depression.. Psychiatry Res.

[30] Nobuhara K, Okugawa G, Minami T, Takase K, Yoshida T (2004). Effects of electroconvulsive therapy on frontal white matter in late-life depression: a diffusion tensor imaging study.. Neuropsychobiology.

[31] Basser PJ, Pierpaoli C (1996). Microstructural and physiological features of tissues elucidated by quantitative-diffusion-tensor MRI.. J Magn Reson B.

[32] Basser PJ, Pierpaoli C (1998). A simplified method to measure the diffusion tensor from seven MR images.. Magn Reson Med.

[33] Kubicki M, Westin CF, Nestor PG, Wible CG, Frumin M (2003). Cingulate fasciculus integrity disruption in schizophrenia: a magnetic resonance diffusion tensor imaging study.. Biol Psychiatry.

[34] Acosta-Cabronero J, Williams GB, Pengas G, Nestor PJ (2010). Absolute diffusivities define the landscape of white matter degeneration in Alzheimer’s disease.. Brain.

[35] Smith SM, Jenkinson M, Johansen-Berg H, Rueckert D, Nichols TE (2006). Tract-based spatial statistics: voxelwise analysis of multi-subject diffusion data.. Neuroimage.

[36] Woolrich MW, Jbabdi S, Patenaude B, Chappell M, Makni S (2009). Bayesian analysis of neuroimaging data in FSL.. Neuroimage.

[37] Smith SM, Jenkinson M, Woolrich MW, Beckmann CF, Behrens TE (2004). Advances in functional and structural MR image analysis and implementation as FSL.. Neuroimage.

[38] Shimony JS, Sheline YI, D’Angelo G, Epstein AA, Benzinger TL (2009). Diffuse microstructural abnormalities of normal-appearing white matter in late life depression: a diffusion tensor imaging study.. Biol Psychiatry.

[39] Li L, Ma N, Li Z, Tan L, Liu J (2007). Prefrontal white matter abnormalities in young adult with major depressive disorder: a diffusion tensor imaging study.. Brain Res.

[40] Wu F, Tang Y, Xu K, Kong L, Sun W (2011). Whiter matter abnormalities in medication-naive subjects with a single short-duration episode of major depressive disorder.. Psychiatry Res.

[41] Colloby SJ, Firbank MJ, Thomas AJ, Vasudev A, Parry SW (2011). White matter changes in late-life depression: A diffusion tensor imaging study.. J Affect Disord.

[42] Makris N, Kennedy DN, McInerney S, Sorensen AG, Wang R (2005). Segmentation of subcomponents within the superior longitudinal fascicle in humans: a quantitative, in vivo, DT-MRI study.. Cereb Cortex.

[43] Schmahmann JD, Pandya DN, Wang R, Dai G, D’Arceuil HE (2007). Association fibre pathways of the brain: parallel observations from diffusion spectrum imaging and autoradiography.. Brain.

[44] Alexander GE, DeLong MR, Strick PL (1986). Parallel organization of functionally segregated circuits linking basal ganglia and cortex.. Annu Rev Neurosci.

[45] Tekin S, Cummings JL (2002). Frontal-subcortical neuronal circuits and clinical neuropsychiatry: an update.. J Psychosom Res.

[46] Dannlowski U, Ohrmann P, Konrad C, Domschke K, Bauer J (2009). Reduced amygdala-prefrontal coupling in major depression: association with MAOA genotype and illness severity.. Int J Neuropsychopharmacol.

[47] Bonelli RM, Cummings JL (2007). Frontal-subcortical circuitry and behavior.. Dialogues Clin Neurosci.

[48] Reetz K, Lencer R, Steinlechner S, Gaser C, Hagenah J (2008). Limbic and frontal cortical degeneration is associated with psychiatric symptoms in PINK1 mutation carriers.. Biol Psychiatry.

[49] Nofzinger EA, Buysse DJ, Germain A, Carter C, Luna B (2004). Increased activation of anterior paralimbic and executive cortex from waking to rapid eye movement sleep in depression.. Arch Gen Psychiatry.

[50] Marchand WR, Lee JN, Johnson S, Thatcher J, Gale P (2012). Striatal and cortical midline circuits in major depression: Implications for suicide and symptom expression.. Prog Neuropsychopharmacol Biol Psychiatry.

[51] Cardoso EF, Fregni F, Martins Maia F, Boggio PS, Luis Myczkowski M (2008). rTMS treatment for depression in Parkinson’s disease increases BOLD responses in the left prefrontal cortex.. Int J Neuropsychopharmacol.

[52] Jhanwar VG, Bishnoi RJ, Jhanwar MR (2011). Utility of repetitive transcranial stimulation as an augmenting treatment method in treatment-resistant depression.. Indian J Psychol Med.

[53] Wall CA, Croarkin PE, Sim LA, Husain MM, Janicak PG (2011). Adjunctive use of repetitive transcranial magnetic stimulation in depressed adolescents: a prospective, open pilot study.. J Clin Psychiatry.

[54] Mayberg HS, Silva JA, Brannan SK, Tekell JL, Mahurin RK (2002). The functional neuroanatomy of the placebo effect.. Am J Psychiatry.

[55] Breiter HC, Rosen BR (1999). Functional magnetic resonance imaging of brain reward circuitry in the human.. Annals of the New York Academy of Sciences.

[56] Haber SN, Fudge JL, McFarland NR (2000). Striatonigrostriatal pathways in primates form an ascending spiral from the shell to the dorsolateral striatum.. The Journal of neuroscience : the official journal of the Society for Neuroscience.

[57] Roitman MF, Wheeler RA, Carelli RM (2005). Nucleus accumbens neurons are innately tuned for rewarding and aversive taste stimuli, encode their predictors, and are linked to motor output.. Neuron.

[58] Blood AJ, Iosifescu DV, Makris N, Perlis RH, Kennedy DN (2010). Microstructural abnormalities in subcortical reward circuitry of subjects with major depressive disorder.. PloS one.

[59] Purves D, Augustine GJ, Fitzpatrick D (2004). Neuroscience: Sinauer Associates; 3rd edition (June.

[60] Brunoni AR, Teng CT, Correa C, Imamura M, Brasil-Neto JP (2010). Neuromodulation approaches for the treatment of major depression: challenges and recommendations from a working group meeting.. Arquivos de neuro-psiquiatria.

[61] Grimm S, Beck J, Schuepbach D, Hell D, Boesiger P (2008). Imbalance between left and right dorsolateral prefrontal cortex in major depression is linked to negative emotional judgment: an fMRI study in severe major depressive disorder.. Biol Psychiatry.

[62] Koenigs M, Grafman J (2009). The functional neuroanatomy of depression: distinct roles for ventromedial and dorsolateral prefrontal cortex.. Behav Brain Res.

[63] Nolen-Hoeksema S, Wisco B, Lyubomirsky S (2008). Rethinking rumination.. Perspect Psychol Sci.

[64] Miranda R, Nolen-Hoeksema S (2007). Brooding and reflection: rumination predicts suicidal ideation at 1-year follow-up in a community sample.. Behav Res Ther.

[65] Bagby RM, Rector NA, Bacchiochi JR, McBride C (2004). The Stability of the Response Styles Questionnaire Rumination Scale in a Sample of Patients with Major Depression.. Cognit Ther Res.

[66] Nolen-Hoeksema S (2000). The role of rumination in depressive disorders and mixed anxiety/depressive symptoms.. J Abnorm Psychol.

[67] Beck AT, Beamesderfer A (1974). Assessment of depression: the depression inventory.. Mod Probl Pharmacopsychiatry.

[68] Bech P (2002). The Bech-Rafaelsen Melancholia Scale (MES) in clinical trials of therapies in depressive disorders: a 20-year review of its use as outcome measure.. Acta psychiatrica Scandinavica.

[69] Peng H, Zheng H, Li L, Liu J, Zhang Y (2012). High-frequency rTMS treatment increases white matter FA in the left middle frontal gyrus in young patients with treatment-resistant depression.. J Affect Disord.

[70] Dalby RB, Frandsen J, Chakravarty MM, Ahdidan J, Sorensen L (2010). Depression severity is correlated to the integrity of white matter fiber tracts in late-onset major depression.. Psychiatry research.

[71] Oudega ML, van Exel E, Wattjes MP, Comijs HC, Scheltens P (2011). White matter hyperintensities, medial temporal lobe atrophy, cortical atrophy, and response to electroconvulsive therapy in severely depressed elderly patients.. J Clin Psychiatry.

[72] Schneider B, Prvulovic D, Oertel-Knochel V, Knochel C, Reinke B (2011). Biomarkers for major depression and its delineation from neurodegenerative disorders.. Prog Neurobiol.

[73] Ladouceura CD, Peperb JS, Croneb EA, Dahlc RE (2011). White matter development in adolescence: The influence of puberty and implications for affective disorders.. Dev Cogn Neurosci.

